# The Use of Physiotherapy in the Conservative Treatment of Cubital Tunnel Syndrome: A Critical Review of the Literature

**DOI:** 10.3390/diagnostics14111201

**Published:** 2024-06-06

**Authors:** Michał Wieczorek, Rafał Gnat, Tomasz Wolny

**Affiliations:** 1 Department of Neurological Rehabilitation, The Health Center in Mikołów Ltd., Waryńskiego 2, 43-190 Mikołów, Poland; 2 Institute of Physiotherapy and Health Sciences, The Jerzy Kukuczka Academy of Physical Education, Mikołowska 72A, 40-065 Katowice, Poland; r.gnat@awf.katowice.com; 3 Institute of Physiotherapy and Health Sciences, Musculoskeletal Elastography and Ultrasonography Laboratory, The Jerzy Kukuczka Academy of Physical Education, Mikołowska 72A, 40-065 Katowice, Poland; t.wolny@awf.katowice.com

**Keywords:** cubital tunnel syndrome, physiotherapy, conservative treatment

## Abstract

Background: The lack of a clear answer regarding the efficacy of physiotherapy in the treatment of cubital tunnel syndrome (CuTS) has led to attempts to critically assess the scientific studies conducted to date. Materials and Methods: Two databases (MEDLINE via PubMed and PEDro) and Google Scholar were used to search for papers. The inclusion criteria were randomized controlled trials, case series, and case reports that evaluate the effects of physiotherapy in the treatment of patients with CuTS. Results: A total of 18 studies met the eligibility criteria, capturing a total of 425 participants. Seven papers were randomized controlled trials, three more described prospective studies without a control group, and eight papers contained case reports. An analysis of the literature evaluating the effectiveness of various forms of broadly defined physiotherapy indicates that their use can have a beneficial effect in reducing many subjective and objective symptoms and improving function. In the majority of papers included in this review, their authors indicated positive therapeutic effects. Only one randomized controlled trial reported no change following therapy. It can therefore be stated that the results of the research conducted so far are optimistic. However, only 7 of the 18 papers were randomized controlled trials, while 3 were prospective studies, and 8 papers were case studies, in which 23 people with CuTS were studied. Conclusions: The small number of randomized clinical trials and their considerable heterogeneity do not allow firm conclusions to be drawn about the effectiveness of physiotherapy in the conservative treatment of CuTS.

## 1. Introduction

Cubital tunnel syndrome (CuTS) is the second most common upper limb peripheral neuropathy in clinical practice after carpal tunnel syndrome [1,2]. The incidence of CuTS is estimated at an average of 21–24 cases per 100,000 patients per year [3] (2–6% in the general population) [4]. The typical clinical picture is characterized by sensory abnormalities in the early phase of the condition in the form of paresthesia and mild hypoesthesia, occurring paroxysmally and mainly related to the position of the elbow. Over time, sensory symptoms worsen, and motor disturbances gradually appear (weakness and atrophy of the intrinsic muscles of the hand) [5,6], which can lead to permanent sensory disturbances, paresis, and joint contractures [7]. McGowan identified three stages of CuTS related to the severity of symptoms, ranging from paroxysmal subjective sensory symptoms (Stage 1), significant sensory loss and weakness of the intrinsic muscles of the hand (Stage 2), to severe sensorimotor deficits with muscle atrophy (Stage 3) [8]. Failure to provide immediate and correct clinical diagnosis and appropriate timely treatment can result in significant impairment of hand function [9]. This, in turn, can have a significant impact on activities of daily living, work, and perceptions of overall quality of health, which is not only a medical problem but also a social and economic one [10].

The diagnosis of CuTS is based on the medical history, physical examination, electrophysiological assessment, and ultrasound examination [9]. However, with anatomical differences, a wide spectrum of symptoms, and the need to differentiate this problem from other neuropathies of the ulnar nerve, diagnosis often poses many problems [9]. Forms of CuTS are often divided into primary (idiopathic) and secondary (symptomatic) [11]. In the case of idiopathic CuTS, a specific morphological cause cannot be determined, making a correct diagnosis difficult [11]. In the secondary form, on the other hand, there are multiple causes (trauma, degenerative changes, cysts, ganglions, etc.) [1,12] and multiple sites of compression of the ulnar nerve (arcade of Struthers, the medial intermuscular septum, the medial epicondyle, the cubital tunnel, and the deep flexor-pronator aponeurosis) [13]. Such a variety of causes, sites of compression, and sometimes a lack of a tangible cause makes it significantly more difficult not only to diagnose but also to choose the most optimal form of therapy.

Both conservative and surgical approaches used in the treatment of CuTS [14]. Conservative treatment is recommended in mild to moderate forms of CuTS [6]. This usually includes pharmacological treatment [15,16], immobilization with elbow night splints [17], and education of the patient on how to change their habits at work and activities of daily living, with particular emphasis on avoiding prolonged elbow flexion [16]. Physiotherapy is also often implemented and includes manual therapy with neurodynamic techniques [18,19], short wave diathermy [20], low-level laser therapy [21], extracorporeal shock wave therapy [22], and even dry needling [23]. Unfortunately, as reported by Mezian et al. [9], conservative treatment of CuTS is based more on empirical experience than on a significant amount of high-quality scientific research. Surgical intervention is used in cases of advanced CuTS or when conservative treatment has failed. The most commonly used surgical methods are simple decompression [24], endoscopic techniques [25], medial epicondylectomy [26], or one of the anterior transposition methods: subcutaneous [27], intramuscular [28], or submuscular [29]. However, there are no scientific reports indicating that any of these techniques can produce better results than the others [30,31]. Palmer and Hughs emphasized that, to date, a ‘gold standard’ in the surgical treatment of CuTS has not been developed [1]. Therefore, there is a lack of scientifically supported treatments for CuTS. This concerns both surgical and conservative treatment, including physiotherapeutic management. Few randomized controlled trials have examined the effectiveness of physiotherapy in CuTS [19,20,21] and the results and conclusions fail to unequivocally demonstrate the beneficial effects of the various physiotherapy methods. Other studies are mostly case reports or case series with a low number of patients studied [18,22,23], which have shown an improvement in the patient’s condition but had a low level of reliability (they are not randomized controlled trials) and should therefore be approached with caution. To the best of our knowledge, there are no recommendations for the physiotherapeutic management of CuTS that are supported by strong scientific evidence. The lack of a clear answer regarding the efficacy of physiotherapy in the treatment of CuTS leads to the attempts to critically assess the scientific studies conducted to date on the use of various forms of physiotherapy in the conservative treatment of this neuropathy. This will help to identify areas that need such research and to deepen or extend the scientific research on the possibility of using different forms of physiotherapy in the conservative treatment of CuTS.

## 2. Materials and Methods

The literature review was conducted in December 2023. Two electronic databases (MEDLINE via PubMed and PEDro) and the Google Scholar search engine were used to search for papers. The search strategy for database papers was based on the use of key phrases and/or abbreviations, taking into account their respective English wording. The following terms were used: cubital tunnel syndrome, CuTS, physiotherapy, physical therapy, PT, electrostimulation, ES, ultrasound, US, magnet therapy, MT, shock wave therapy, RSW, shortwave diathermy, laser, neuromobilization, neurodynamic techniques, neurodynamic treatment, neurodynamic mobilization, nerve gliding exercises, neurodynamics, manual therapy, conservative treatment, and splitting.

Studies that met the following criteria were included in the review: (1) the participant had clinically and/or electrophysiologically diagnosed CuTS, and (2) any form of physical stimulus was used in the study. All papers were accepted regardless of the year of publication. The search was limited to papers published in English.

**Figure 1:**
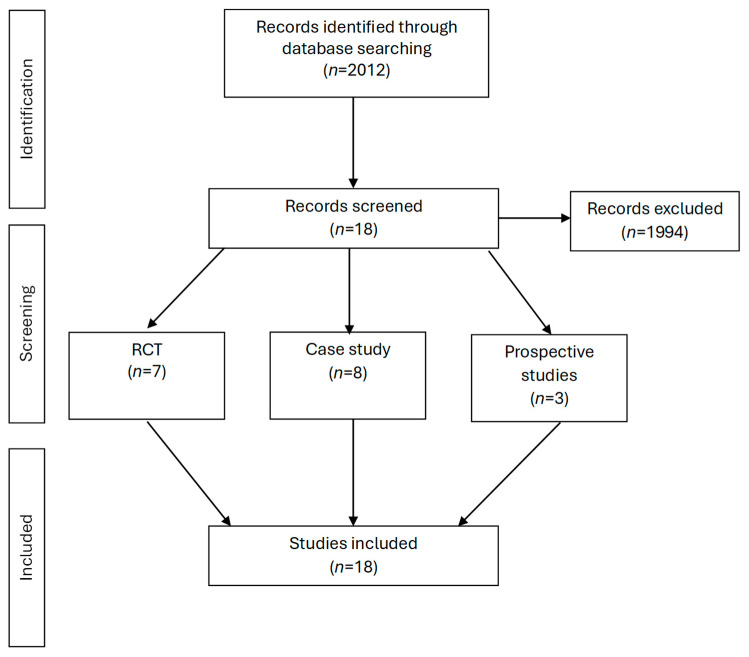
Flow charts.

The titles and abstracts of the retrieved papers were analyzed to assess whether they met the inclusion criteria. Those that did not show relevance to the subject area studied were excluded, e.g., those that contain surgical treatment approaches. The bibliographies of the included papers were also analyzed to check whether other papers meeting the criteria had not been omitted (Figure 1).

## 3. Results

Eighteen papers met the inclusion criteria [17,18,19,20,21,22,23,32,33,34,35,36,37,38,39,40,41,42]. Seven papers were randomized controlled trials [19,20,21,32,33,40,42] (Table 1), three more described prospective studies without a control group [17,37,39] (Table 2), and eight papers contained case reports [18,22,23,34,35,36,38,41] (Table 3). In six papers, the experiments were based on two comparison groups [20,21,32,33,40,42]; in one paper there were three groups [19]; three had no comparison groups [17,37,39]; and eight contained only case descriptions [18,22,23,34,35,36,38,41]. Participants were examined before the therapy in all the reviewed studies, and immediately after therapy in ten studies [20,21,32,34,35,36,37,38,39,42]. The long-term effects were assessed in thirteen studies [17,18,19,20,21,22,23,33,36,38,39,40,41] and at different follow-up times, ranging from two weeks to twelve months. In two studies, participants were assessed before each therapy session [18,23]. Also, two studies evaluated participants during the study [37,39]. In only one case, the study was double-blinded [20], one study was single-blinded [21], and two studies investigated the placebo effect [20,40]. There is no information in the other studies about their blinding [17,18,19,22,23,32,33,34,35,36,37,38,39,41,42].

Seventeen papers had participants clinically and/or electrophysiologically diagnosed with CuTS, while one paper did not state how the diagnosis was made [42]. The number of participants ranged from 1 to 77. The age of the subjects was documented in seventeen papers [17,18,19,20,21,22,23,32,33,34,35,36,37,38,40,41,42] and ranged from seventeen to seventy-nine years. The interventions were described in men and women in nine papers [17,19,20,21,23,37,39,40,42], in women in four papers [18,32,34,35], and in men in three papers [33,36,41]. Two papers did not specify the gender of patients receiving treatment [22,38].

### 3.1. RCT Studies

In the manuscripts describing the randomized controlled trials, the most frequent assessments concerned pain severity (VAS) (five studies) [19,20,21,32,40] and grip strength (five studies) [19,20,21,32,42]. Four studies performed neurophysiological assessment (NCS) [19,21,33,40] while three assessed hand function using the DASH or QuickDASH questionnaires [20,32,40]. Three others used the Tinel’s test for assessment [20,21,32]. Assessment of activity (COMP) [19], neurological signs and symptoms [33], adduction of the little finger [19], pincer grip strength [32], muscle strength (MRC) [20], CuTS severity according to McGowan [40], quality of life using the SF-36 questionnaire [20], patient satisfaction [21], sensory threshold [21], neuropathy (SQUNE) [40], and neuromuscular ultrasound (NMUS) [40] were each used only once. Elbow night splinting [19,33,42], neurodynamic techniques [19,32,42], and ultrasound therapy [21,32,42] were evaluated in at least one of the four papers, either as the sole form of intervention [19,21,32,42] or as a component of the complex treatment applied [32,33,42]. Two studies used sham therapy to assess the effect of placebo on treatment [20,40]. The following physical stimuli were used in at least one study: short-wave diathermy [20], low-level laser [21], shock wave [40], and dry cupping [32]. Elements of treatment used in studies included strengthening exercises, autoneuromobilization, and co-contraction in one study [32], as well as patient education [19] and steroid injection [33]. Intra-group improvements, with no significant intergroup differences, were found in four studies [19,21,32,33], while intergroup differences after the interventions were observed in two papers [40,42], and one study found no changes in the parameters measured [20].

### 3.2. Prospective Studies

In the papers describing prospective studies without a control group, the following methods were used to assess the condition of the participants: two-point discrimination assessment [17,37,39], sensory threshold assessment based on Semmes–Weinstein monofilament [17,37], neurophysiological assessment (NCS) [17,37,39], Tinel’s sign [17,39], elbow flexion provocation test [39], measurements of grip strength and pinch grip strength [37,39], measurement of global muscle strength (MMT) [37], strength assessment of the first dorsal interosseous and flexor digitorum profundus to small finger [39], pain assessment (VAS) [17], assessment using the QDASH questionnaire [39], and SF-12 [39]. All of these papers described elbow night splinting as a form of intervention [17,37,39], and two additionally instructed participants on how to modify their activities of daily living [37,39]. Furthermore, a significant proportion of participants improved on all assessed parameters (73% to 100%) [17,37,39].

### 3.3. Case Studies

The case reports used a variety of methods to assess patients conditions, including pain severity score (VAS, NRS) [18,23,35,36,38], and measurements of grip strength [23,34,38] and pincer grip strength [38]. The sensory threshold was also assessed [38]. Provocative tests, such as elbow joint flexion [18,35,38], Tinel’s sign [18,34,38], the elevated arm stress test (EAST) [34], and the nerve provocation test [18,35,41] were carried out. Structural changes were assessed (PAM) [35] and a selective tissue tension test (STTT) was performed [35]. The DASH or QuickDASH questionnaires [22,38,41] were used to assess upper limb function, and, in one study, participants self-assessed neuropathic symptoms (S-LANSS) [41]. The global rating of change (GROC) [23,41] and patient-specific functional scale (PSFS) [23] were also employed. Furthermore, the severity of paresthesias [22,34], range of motion (ROM) [18,35], and functional status (NPQ) [18] were measured. Neurophysiological examination was performed in three cases (NCS) [18,22,36], and magnetic resonance imaging (MRI) [36] was conducted in one study. Neurodynamic techniques were used as treatment methods [18,38,41], and in one of the studies, it was performed by the patient [41]. Ultrasound therapy [38], shock wave [22], pulsed radiofrequency (PRF) [36], percutaneous electrical stimulation (PENS) [41], manual therapy [35], chiropractic therapy [34], dry needling [23], kinesiotaping [34], cold packs [38], and strengthening and resistance exercises [38] were also used. In all the studies cited, the authors achieved significant improvements and/or resolution of complaints [18,22,23,34,35,36,38,41].

## 4. Discussion

An analysis of the literature evaluating the effectiveness of various forms of broadly defined physiotherapy in the conservative treatment of CuTS indicates that their use can have a beneficial effect in reducing many subjective and objective symptoms and improving function. In the vast majority of papers included in this review (17/18), their authors indicated positive therapeutic effects [17,18,19,21,22,23,32,33,34,35,36,37,38,39,40,41,42]. Only one randomized controlled trial reported no change following therapy compared with a sham control group [20]. It can therefore be stated that the results of the research conducted so far are optimistic. However, it seems that the results obtained and the conclusions drawn from them should also be viewed critically. Only 7 of the 18 (39%) papers included in the review were randomized controlled trials [19,20,21,32,33,40,42], while 3 (17%) were prospective studies without a control group [17,37,39], and 8 (44%) papers were case studies [18,22,23,34,35,36,38,41], in which 23 people with CuTS were studied.

Randomized controlled trials have the lowest risk of bias, so they provide the most objective and reliable results. Most of the randomized controlled trials included in this review reported beneficial effects of the therapy (in 6/7 papers) [19,21,32,33,40,42]. However, it should be emphasized that in only four papers [19,21,33,40] the diagnosis of CuTS was based on medical history, orthopedic examination, and electrophysiological examination, which is considered a ‘gold standard’ in the diagnosis of compression syndromes [43]. In other studies, the diagnosis was based only on the medical history and orthopedic examination [20,32,42], which, of course, is very often sufficient in combination with an ultrasound examination, but, without an ultrasound and electrophysiological examination, other causes cannot be excluded, such as Guyon canal syndrome, cervical radiculopathy, or brachial plexopathy, which can produce similar symptoms [9]. Special attention should also be paid to the different methodologies used in all eligible randomized controlled trials. The majority of the papers assessed pain complaints (5/7 papers) [19,20,21,32,40], but although pain is the predominant symptom, it is not necessarily specific to CuTS. While several studies investigated muscle strength [19,20,21,32,42], function [20,32,40], and sensation [21], only one analyzed the overall quality of health [20]. The large spectrum of parameters studied makes it significantly more difficult to compare the results obtained from different studies. A similar observation can be made for the multitude of different physiotherapy interventions. In fact, different physiotherapy programs were used in each paper selected for review. In three out of seven papers [21,32,42], one of the groups used ultrasounds as one of the components of physiotherapy programs, and in two papers, it was used as the only form of therapy [21,42]. Also, in three papers, the common element of the physiotherapy program was neurodynamic techniques [19,32,42], but the other components of the physiotherapy program were different. Neurodynamic mobilizations to enhance ulnar nerve gliding include sliding techniques and, to a lesser extent, tensioning techniques. A sliding technique is an alternation of combined movements of (at least) two joints in which one movement loads the peripheral nervous system while the other movement simultaneously unloads the nervous system. These techniques facilitate nerve gliding without intensely challenging the nervous system. This process allows gliding to take place inside the nerve itself and between the surrounding tissues. The patient is in a supine position with a contralateral side bending position of the neck. External rotation is 90° abduction, and the shoulder is positioned in depression. The therapist executes passive forearm pronation and supination whereas the elbow is flexed to 90°. In the second step, the therapist pronates the forearm and conducts passive elbow flexion from 90° to 140° from the first position. Third, the therapist conducts shoulder depression from 90° to 120° of abduction, beginning from the final position obtained with the elbow in 90° flexion [18,32,42]. Also, in three papers, limb immobilization with a splint was used as the only therapy or as one of the components of a therapy program [19,33,42]. Other studies used short-wave diathermy [20], laser [21], shock wave [40], and strengthening exercises [32].

Benincá et al. [44] describe diathermy as the use of high-frequency electromagnetic currents to induce heat. It has many applications, such as producing tissue damage during surgery, hyperthermia treatment in oncology, and as a deep heating method in rehabilitation. The heating effects include pain relief, increased nerve conduction velocity, blood flow, local metabolism, tissue elasticity, and muscle relaxation. One form of diathermy used by physical therapists that achieves these effects is shortwave diathermy (SWD), which produces shortwave electric and magnetic fields in the tissue. SWD can be delivered by an inductive technique, in which the greatest amount of heat occurs in deep tissue, or by a capacitive technique, in which the greatest amount of heat occurs in superficial tissue. Inductive types of electrodes consist of a current flowing through a coiled cable that may be contained within drum or sleeve electrodes. Capacitive electrodes consist of two air plates or pad electrodes that allow for three arrangements: coplanar (electrodes placed side by side, tissues parallel to the current), anti-planar (electrodes on either side of the body part, tissues in series to the current), or longitudinal (electrodes placed at each end of the limb on opposite sides of the body part, tissues in series to the current). Each technique can be continuous (CSWD) or pulsed (PSWD), the continuous mode being used primarily for thermal effects, while the pulsed mode is used primarily for non-thermal effects.

Low-level laser therapy (LLLT) is described by Glazov et al. [45] as a light source treatment that may act via non-thermal or photochemical reactions in cells. It includes laser acupuncture, which involves focused irradiation at specific points, most commonly traditional acupuncture points, with a low-intensity laser. LLLT for pain relief in medicine remains controversial with claims that apparent efficacy is due to the placebo effect. Multiple mechanisms for LLLT analgesia may exist. There is experimental evidence suggesting that laser irradiation induces peripheral neural blockade, suppresses central synaptic activity, modulates neurotransmitters, reduces muscle spasm and interstitial oedema, and exerts anti-inflammatory effects.

Extracorporeal shock wave therapy (ESWT) was described by Yao et al. [46] as a non-invasive procedure in which acoustic waves are focused on targeted sites within the body to facilitate pain relief and healing. In general, ESWT is considered safe, non-invasive, easy to apply, and well tolerated by most patients, and so has been widely used for many musculoskeletal conditions over the last 25–30 years. Although the exact mechanisms for analgesic and functional effects are still incompletely understood, it is suggested that shock waves accelerate tissue regeneration, reduce calcification, and inhibit pain receptors.

This wide variation in the therapeutic programs in the randomized clinical trials conducted to date considerably complicates the comparison of study results. Therefore, the results obtained should be viewed with some caution. It should also be emphasized that only two out of seven randomized clinical trials performed a longitudinal evaluation of the effectiveness of the applied physiotherapy program [19,33], which indicated the achieved effects of the applied therapy were maintained after six months. The remaining five studies evaluated the effectiveness of the therapy program directly [32,42] or a maximum of 3 months [20,21,40] after its completion, so it cannot be assumed that the effects would be sustained over a longer period of time, and it seems that maintaining sustained improvements is most important from the point of view of its effectiveness.

In all three prospective studies without a control group included in this review, some improvements were obtained in patients with CuTS [17,37,39]. Improvements were documented in all participants in the first study [17], 88% of participants in the second [39], and 73% in the third [37]. Neurophysiological assessment was also performed in all studies, although subjects with normal nerve conduction who showed typical CuTS symptoms were also included [39]. Sensory threshold and discriminative sensation were assessed in two studies [17,37], while only discriminative sensation was assessed in one study [39], and pain intensity was assessed in one study [17]. Two out of three papers measured muscle strength [37,39], and one study evaluated the degree of disability [39] and general quality of health [39]. In all three studies, the main component of therapy was an elbow night splinting [17,37,39]. Two studies employed additionally provided instruction on changing habits during activities of daily living. All studies examined not only the effects immediately after the therapy but also the long-term effects one year later [17,37,39]. Although orthoses were used in all of these studies, unfortunately, the angle of flexion varied in each study (from the elbow in full extension to flexion up to 60°), which was not justified in any way. Unfortunately, the main limitation of all these studies was the lack of a control group. Therefore, it is impossible to verify whether the effects obtained result from the therapy used, spontaneous recovery, or the placebo effect.

Positive effects of the applied therapy were obtained in all case studies [18,22,23,34,35,36,38,41]. The studies reported a reduction in pain [18,22,23,36,38,41], improved function [18,22,23,38,41], increased muscle strength [23,34,38], and reduced symptoms in provocation tests [18,34,35,38]. Evaluation of therapeutic effects occurred immediately after therapy but in 6/8 papers also much later: 3 [22,36,41], 6 [18,23,36,41], and even 12 months [38,41] after the intervention. Unfortunately, the small number of study participants (23 people in all studies), the very large methodological variation, and the use of different physiotherapy programs require considerable caution in drawing conclusions about the effectiveness of the therapy used. The studies assessed pain [18,23,35,36,38], range of motion [18,35], sensation [38], muscle strength [34,38], and hand dexterity [22,38,41], conducted provocation tests [18,34,35,38]. Therapeutic interventions varied from neurodynamic techniques, spinal and rib manipulation [18], manual therapy [35], electric therapy [36,41], ultrasound [38], shock wave [22], and dry needling [23]. Dry needling as described by Gattie et al. [47] is a technique in which a fine needle is used to penetrate the skin, subcutaneous tissues, and muscle with the intention of mechanically disrupting the tissue without the use of an anesthetic. Dry needling is often used to treat myofascial trigger points (MTrPs), which are described as localized hypersensitive areas within a palpable taut band of muscle. These hypersensitive areas can be classified as active MTrPs when they elicit spontaneous pain and, on palpation, reproduce the pain known to the patient. Latent MTrPs do not elicit spontaneous pain and are painful only on palpation. Myofascial trigger points are commonly seen in patients with musculoskeletal pain. The physiologic mechanism underlying the action of dry needling remains to be elucidated. However, it has been suggested that dry needling may induce both local and central neural responses to restore homeostasis at the MTrP site, resulting in a reduction in both peripheral and central pain sensitivity. Centrally, dry needling may activate descending control mechanisms in the brain or spinal cord. Dry needling has been shown to immediately increase pressure pain threshold and range of motion, reduce muscle tension, and reduce pain in patients with musculoskeletal disorders.

The number of therapy sessions also varied and equaled 1 [36], 3 [41], 5 [18], or 11 [34]. Unfortunately, in the vast majority of these papers, the diagnosis of CuTS was based only on medical history and/or orthopedic examination [18,22,23,34,35,38,41], while electrophysiological examination was performed in only three cases [18,22,36], including one that was within normal values [18]. It is therefore difficult to confirm whether in all case studies the patients actually had CuTS or whether the symptoms had another cause. Also, in case study research, the lack of comparison with non-therapy cases may indicate that the effects obtained after therapy are the result of spontaneous recovery or a placebo effect. It should also be emphasized that case studies are subjected to a high risk of bias, and therefore the results obtained from the physiotherapy programs used should be approached with caution.

Techniques that may be useful in treating CuTS include interferential current (IFC), transcutaneous electrical nerve stimulation (TENS), neuromuscular electrical stimulation (NMES), and deep oscillation procedures. According to Rampazo and Liebano [48], IFC therapy involves the transcutaneous application of two medium-frequency alternating currents (>1 kHz to <10 kHz) out of phase to deliver current to deep tissues. For example, one of the alternating currents may have a fixed frequency of 4000 Hz, while the frequency of the other alternating current may be set between 4000 and 4250 Hz. The two medium-frequency currents “interfere” in the tissue and produce an amplitude-modulated low-frequency “beat” (0–250 Hz) which is the difference between the values of the two applied currents. IFC is an example of a pulse-modulated sinusoidal alternating current, also known as kilohertz alternating current, and is reportedly more comfortable, reaches deeper tissues, and produces greater muscle torque than low-frequency pulsed currents. There are several parameters that can be adjusted in IFC devices, including the carrier frequency, amplitude modulated frequency, sweep frequency, sweep mode or swing pattern (slope), application type (bipolar or quadripolar application), and application time and intensity.

Mokhtari et al. [49] describe TENS as a noninvasive pain relief technique that activates peripheral nerves by delivering electrical impulses to the intact skin surface, which can modulate the transmission of nerve impulses by inhibiting presynaptic nociceptive information. In practice, TENS can be implemented using different stimulus parameters in terms of frequency, intensity, and electrode placement. Since TENS with different stimulus parameters can activate different populations of nerve fibers, this technique can be divided into different types, including conventional TENS (low intensity and high frequency), acupuncture-like TENS (high intensity and low frequency), and intensive TENS (high intensity and high frequency).

As described by Doucet et al. [50], neuromuscular electrical stimulation, used interchangeably with electrical stimulation (ES), is typically delivered at higher frequencies (20–50 Hz) with the express purpose of producing tetany and muscle contraction that can be used for “functional” purposes. The frequencies of electrical stimulation used can vary widely depending on the goals of the task or intervention, but most clinical regimens use patterns of 20–50 Hz to achieve optimal results. To avoid fatigue or discomfort, a constant low-frequency stimulation that produces a smooth contraction at low force levels is typically used. Electrical stimulation devices deliver pulses in waveforms that are often represented by geometric shapes such as a square, spike, or sine wave. Controversy regarding optimal electrode placement is common in the literature, with most debates centering on whether the muscle belly or the motor point is the preferred location. Program duration has ranged from 30 min once a day to an hour in each session three times daily. The total treatment period ranged from 2 weeks to 3 months.

Deep Oscillation (DO), as described by Villalba-Meneses et al. [51], is a patented manual therapy that produces mechanical vibrations in the skin and deep tissues. This device uses repeated electrostatic oscillations to relieve the pain and swelling of a specific area by moving the swelling through the lymphatic system. DO therapy builds up a pulsating electrostatic field of low intensity (100–400 V; 150 μA) and low frequency (5–250 Hz) between the hand applicator and the affected tissue. Its low-frequency electrostatic field produces a throbbing effect in the underlying tissues that improves wound healing and anti-inflammatory effects, stimulates lymphatic flow, stimulates collagen production, and cell regeneration, and ensures more blood reaches the affected area. Both the patient and the physical therapist are connected to the deep oscillation device, which serves as a source of tension with high internal resistance. The impulse of the voltage produces an electrostatic attraction on the tissue and rhythmic frictions are generated when massaging the oedema. These rhythmic frictions result in oscillations of the local tissue (skin, conductive tissue, subcutaneous adipose tissue, muscles, blood, and lymphatic vessels) and increase the vascular circulation of the area concerned. Each session of deep oscillation therapy follows a rigorous clinical protocol. In most medical trials, therapy begins with 15 min of conventional manual lymphatic drainage. Later, the DO equipment is used for 10 min at 200–250 Hz in the area to be treated, and oscillations of 85 Hz are administered for 10 min to finish. The physiological effects of deep oscillation therapy in the treatment of low back pain will depend on the frequency and intensity applied. Clinical studies have reported that DO therapy restores mobility between fibers, repairs affected tissue, exerts anti-inflammatory effects, reduces oedema, improves drainage channels, and accelerates wound healing.

It must be emphasized that, from the broader perspective, studies to date on the effectiveness of physiotherapy in the conservative treatment of CuTS have found beneficial effects, although no clear far-reaching conclusions can be drawn at this stage. This is mainly due to the small number of randomized clinical trials, the significant methodological discrepancies regarding both the diagnosis of CuTS and the parameters studied, and the significant variety of therapeutic programs used. Therefore, high-quality randomized controlled trials evaluating the effectiveness of different physiotherapy measures in the conservative treatment of CuTS are needed. Due to the frequent occurrence of CuTS, case studies do not seem to be relevant. Since positive therapeutic effects were documented in most of the papers included in this review, similar therapeutic programs based on manual therapy incorporating neurodynamic techniques, ultrasound therapy, low-level laser, or other measures that had a beneficial effect on the patient’s condition could be used in further studies. It would also be advisable to conduct studies with blinding of participants and investigators.

This critical review of studies evaluating the effectiveness of physiotherapy in the conservative treatment of CuTS has some limitations. Only two databases and the Google Scholar search engine were used to search for papers for the review. This was limited to English-language papers only, so other valuable studies might have been likely omitted, especially randomized controlled trials that have evaluated the effectiveness of physiotherapy in the conservative treatment of CuTS. Another limitation is that we have only managed to cover a limited number of the existing physiotherapy procedures with positive effects on CuTS.

## 5. Conclusions

A review of studies to date evaluating the effectiveness of physiotherapy in the conservative treatment of CuTS leads to the conclusion that beneficial therapeutic effects were achieved in the majority of these studies. The small number of randomized clinical trials and their considerable heterogeneity do not allow firm conclusions to be drawn about the effectiveness of physiotherapy in the conservative treatment of CuTS.

## 6. Future Directions

High-quality randomized controlled trials evaluating the effectiveness of different physiotherapy programs in the conservative treatment of CuTS are needed.

## Figures and Tables

**Table 1:** Characteristics of randomized clinical trials (RCT).

Study	Participants	Measured Parameters	Control	Intervention	Result
Svernlöv et al. [19]	*n* = 70 sex: 39 female, 31 male age: 17–72 years	Evaluation at baseline and after 6 months. Assessment of activity (COPM), grip strength (JAMAR^®^ dynamometer), adduction strength of the fifth finger (custom measuring devices), pain (VAS), and neurophysiological assessment (NSC, electromyography)	Three groups	Group A—elbow orthosis (for 3 months); Group B—ulnar nerve neuromobilization; Group C—instructed as to the nature of the condition	Significant improvement in all groups, with no statistically significant intergroup differences
Badur et al. [20]	*n* = 61 sex: 32 female, 29 male age: 16–79 years	Assessment at baseline, post-treatment, 1 and 3 months post-treatment. Pain (VAS), Tinel’s sign, arm, shoulder and hand disability questionnaire (QuickDASH), quality of life assessment (SF-36 questionnaire), grip strength assessment (dynamometer), and muscle strength assessment (MRC)	Two groups	Group A—short-wave diathermy (10 treatments), Group B—sham short-wave diathermy, placebo (10 treatments)	No change in assessed parameters in either group
Ozkan et al. [21]	*n* = 32 sex: 16 female, 16 male age: mean = 43.5 years	Assessed at baseline, post-treatment, and 1, 3 months after treatment. Pain (VAS), grip strength (dynamometer), Tinel’s sign, sensory threshold assessment (Semmes–Weinstein monofilament test), neurophysiological assessment (NCS), and patient satisfaction scale	Two groups	Group A—low-level laser therapy (10 treatments); Group B—ultrasound therapy (10 treatments)	Improvement in both groups after the intervention, with no intergroup differences
Alashkar and Hablas [40]	*n* = 47 sex: 10 female, 37 male age: mean = 34.8 years	Assessment at baseline, 2 weeks after the end of treatment, and 3 months after the end of treatment. Pain (VAS), McGowan grade (MGS), self-completed elbow neuropathy evaluation questionnaire (SQUNE), shortened disability questionnaire for arm, shoulder and hand (QuickDASH), neurophysiological assessment (NCS), and neuromuscular ultrasound (NMUS)	Two groups	Group A—shock wave therapy (3 therapies); Group B—sham shock wave therapy, placebo (3 therapies)	A statistically significant improvement was obtained in favor of the experimental group (Group A)
Garber et al. [42]	*n* = 40 sex: 23 female, 17 male age: mean = 50.6 years	Pre- and post-treatment evaluation. Grip strength (dynamometer)	Two groups	Group A—ultrasound therapy (18 therapies); Group B—ulnar nerve neuromobilization (18 therapies); overnight splint of the elbow in both groups	Greater increase in grip strength was shown in Group B (neuromobilization)
Galal et al. [32]	*n* = 24 sex: female age: mean = 37.4 years	Assessment at baseline and after completion. Pain (VAS), Tinel’s sign, disability questionnaire of the arm, shoulder and hand (DASH), and grip and side grip strength (JAMAR^®^ dynamometer (Lafayette Instrument Company, Lafayette, LA, USA))	Two groups	Group A—ultrasound therapy (18 therapies), ulnar nerve neuromobilization (18 therapies), strengthening exercises with dumbbells, closed chain exercises, co-contraction on a Swiss ball, neurodynamic self-therapy (10 times a day for 6 weeks); Group B—same therapy as Group A, plus dry cupping (18 therapies)	Improvements were observed in all assessed parameters, with no intergroup differences.
Hong et al. [33]	*n* = 10 sex: male age: 37–70 years	Assessment before, and 1 and 6 months after treatment. Assessment of neurological symptoms and signs, and neurophysiological assessment (NCS)	Two groups	Group A—steroid injections, elbow splint overnight; Group B—elbow splint overnight	Improvements were shown in all assessed parameters in both groups, with no intergroup differences

COPM—Canadian Occupational Performance Measure; VAS—Visual Analogue Scale; NCS—Nerve Conduction Study; DASH—Disabilities of the Arm, Shoulder, and Hand Outcome Measure; MRC—Medical Research Council; MGS—McGowan scale; SQUNE—Self-administered Questionnaire of Ulnar Neuropathy at the Elbow; NMUS—Neuromuscular Ultrasonography; SF-36—Short-Form Health Survey with 36 questions.

**Table 2:** Characteristics of papers describing prospective studies without a control group.

Study	Participants	Measured Parameters	Control	Intervention	Result
Seror [17]	*n* = 22 sex: 10 females, 12 males sex: 39–81 years	Evaluation at baseline and after 6 and 12 months. Pain (VAS), provocative test (Tinel’s sign), discrimination sensory assessment, sensory threshold assessment (Semmes–Weinstein monofilament test), and neurophysiological assessment (NCS)	No	Elbow splint overnight between 15° and 60°, without limiting pronation and supination of the forearm	100% of patients achieved improvement in assessed parameters
Shah et al. [39]	*n* = 19 sex: 11 females, 8 males age: no data	Assessment at baseline, at 6 weeks, at 3 months (post), and at 12 months. Disability questionnaire for the arm, shoulder and hand (QuickDASH), quality of life assessment (SF-12 questionnaire), provocative tests (Tinel’s sign, elbow flexion test), presence or absence of Froment’s sign, grip strength assessment (JAMAR^®^ dynamometer), pincer grip strength (dynamometer), muscle strength: first dorsal interosseous and flexor digitorum profundus to small finger (according to BMC), two-point discrimination, and neurophysiological assessment (NCS)	No	Night splint of the elbow in 45° of flexion for a period of 3 months, instruction on changing habits in daily activities (non-irritation of the ulnar nerve)	88% of patients experienced improvement in assessed parameters
Nakamichi et al. [37]	*n* = 77 (80 nerves) sex: 21 females, 8 males age: 19–77 years	Evaluation at the beginning, every 3–4 weeks, and one year after obtaining a plateau. Evaluation of two-point discrimination, sensory threshold (Semmes–Weinstein monofilament test), evaluation of muscle strength (MMT), evaluation of grip strength and pinch grip strength, and neurophysiological evaluation (NSC)	No	Education of patients on the etiology of the disease and the necessity to change habits during daily activities; patients experiencing increased symptoms upon awakening were advised to splint the elbow in an upright position at night	73% of patients reported improvement in assessed parameters

VAS—Visual Analogue Scale; NCS—Nerve Conduction Study; DASH—Disabilities of the Arm, Shoulder, and Hand Outcome Measure; SF-12—Short-Form Health Survey with 12 questions; MMT—Manual Muscle Test; BMC—British Medical Council.

**Table 3:** Characteristics of papers with case reports.

Study	Participants	Measured Parameters	Control	Intervention	Result
Coppieters et al. [18]	*n* = 1 sex: female age: 17 years	Evaluation at baseline, before each treatment session, at 6 and 10 months after the end of treatment. Pain (VAS), range of motion (goniometer), provocation tests (elbow flexion test, Tinel’s sign), ulnar nerve neural provocation test (NPT_UN_), functional status (NPQ), and neurophysiological assessment (NSC)	No	Neurodynamic mobilization (5 treatments), elbow mobilization (4 treatments), home therapy—active ulnar nerve glide (5 treatments), manipulative techniques for Th8-Th9 and ribs 7 and 8 (3 treatments), education (1 instruction)	After therapy, in each of the tests used, the symptoms were eliminated. The effect persisted 10 months after therapy
Kwak et al. [36]	*n* = 2 sex: male age: 39 and 40 years	Evaluation at baseline, after treatment, then at 1, 2, 3, and 6 months after treatment. Pain (NRS), neurophysiological assessment (NCS), elbow and magnetic resonance imaging (MRI)	No	PRF under ultrasound guidance—1 treatment	After 1 treatment session, the pain subsided; evaluation at 1, 2, 3, and 6 months after the end of treatment showed no pain return
Fernández-de-Las-Peñas et al. [41]	*n* = 1 sex: male age: 48 years	Evaluation at baseline and at 1, 3, 6, and 12 months after treatment. Provocative tests (Tinel’s sign, elbow flexion test), neural provocation test (NPT_UN_), disability questionnaire for arm, shoulder and hand (DASH), self-assessment of neuropathic symptoms (S-LANSS), and global rating of changes (GROC)	No	PENS under ultrasound guidance—3 treatments; neuromobilization of the ulnar nerve—performed independently for 2–3 weeks	After 3 treatment sessions, pain was eliminated and functional improvement was achieved; the effect lasted for 12 months
Kearns and Wang [35]	*n* = 1 sex: female age: 45 years	Assessment at the beginning and after treatment. Selective tissue tension test (STTT), range of motion (goniometer), provocative tests (ULTT, elbow flexion test), structural abnormalities (PAM), and pain (NRS)	No	Manual therapy to restore glide in the elbow and wrist (4 therapy sessions)	After four weeks, elimination of symptoms and paresthesia during provocative tests, restoration of normal ranges of motion
Oskay et al. [38]	*n* = 7 sex: no data age: 35–70 years	Evaluation at baseline, at the end of treatment, and 12 months after the end of treatment. Provocative tests (Tinel’s sign, elbow flexion test), measurement of grip strength and pinch grip, pain (VAS), assessment of sensory threshold (Semmes–Weinstein monofilament test); disability of arm, and shoulder and hand questionnaire (DASH)	No	Ultrasound on the tract of the ulnar nerve (10 sessions), neurodynamic mobilization (10 sessions), cold compresses (15 min) were applied after the end of the therapy session; stretching exercises and resistance exercises	Pain and Tinel’s sign were decreased; improved limb function (DASH), increased pincer grip strength and grip strength throughout follow-up period
Shen et al. [22]	*n* = 7 sex: no data age: 35–71 years	Assessment at baseline and at 4, 8, and 12 weeks after the end of treatment. Severity of paresthesia and dysesthesia (VAS), Quick disability questionnaire for arm, shoulder and hand (QuickDASH), and neurophysiological assessment (NCS)	No	Extracorporeal shock wave therapy (3 treatments)	Reduction in VAS and QuickDASH score at the end of treatment and throughout the follow-up period
Anandkumar and Manivasagam [23]	*n* = 3 sex: 2 male and 1 female age: 35, 45, 50 years	Assessment at baseline, before each treatment session, and at 6 months after the end of treatment. Pain (NRS), functional limitations (PSFS), grip strength without pain (JAMAR^®^ dynamometer), and self-reported global rating of change (GROC)	No	Dry needling (2 therapy sessions per week for 2 weeks)	Reduction in pain and return to full functional ability in all patients; in addition, an increase in pain-free grip strength and in self-assessment of changes; the effect persisted for 6 months after the end of treatment
Illes and Johnson [34]	*n* = 1 sex: female age: 41 years	Assessment at the beginning and after treatment. Numbness severity (VAS), provocative tests (Tinel’s sign), arm elevation test (EAST), and grip strength (blood pressure cuff)	No	Chiropractic therapy in the cervical spine (11 therapy sessions), myofascial therapy, kinesiotaping, home exercises (twice a week for 4 weeks), ergonomics of the work environment	After 11 treatment sessions, symptoms resolved completely

NRS—Numeric Rating Scale; PSFS—Patient Specific Functional Scale; GROC—Global Rating of Change; VAS—Visual Analogue Scale; NPQ—Northwick–Park Questionnaire; DASH—Disabilities of the Arm, Shoulder, and Hand Outcome Measure; S-LANSS—Leeds Assessment of Neuropathic Symptoms and Signs; PENS—Percutaneous; Electrical Stimulation; STTT—Selective Tissue Tension Test; ULTT—Upper Limb Tension Test; PAM—Passive Accessory Movement; PRF—Pulsed Radiofrequency; NCS—Nerve Conduction; Study; MRI—Magnetic Resonance Imaging; EAST—Elevated Arm Stress Test; NPT_UN_—Neral Provocation Test, Ulnar Nerve; Th8-Th9—Thoracic Vertebra 8,9.

## Data Availability

Data are available upon request.
